# Implementing population-wide mass media campaigns: Key drivers to meet global recommendations on fruit and vegetable consumption

**DOI:** 10.1371/journal.pone.0273232

**Published:** 2022-08-17

**Authors:** Sirinya Phulkerd, Sasinee Thapsuwan, Aphichat Chamratrithirong, Rossarin Soottipong Gray, Umaporn Pattaravanich, Chantana Ungchusak, Pairoj Saonuam

**Affiliations:** 1 Institute for Population and Social Research, Mahidol University, Nakhon Pathom, Thailand; 2 Healthy Lifestyle Promotion Section of Thai Health promotion Foundation, Bangkok, Thailand; PhD, PLOS, UNITED KINGDOM

## Abstract

While the influence of implementation of mass media and community-based campaigns has been evident elsewhere, this information has been notably absent in Thailand. This study aimed to investigate the association between implementation of mass media campaigns (MMC) and community-based campaigns (CBC) for increased fruit and vegetable (FV) consumption to meet the global recommended level in the Thai population. The sample was obtained from a nationally-representative, longitudinal household survey of the Thai population, Round1 (2018) and Round2 (2019). The study applied a multi-stage sampling design to obtain a sample of persons age 15 years or older. Face-to-face interviews were conducted with 3,010 respondents who successfully participated in both Round1 and Round2 surveys. Information on FV consumption, exposure to Government MMC, ThaiHealth MMC including social marketing (MMSMC) and CBC, and sociodemographic characteristics were collected. Binary logistic regression analysis was used to investigate the association between implementation of those campaigns and increased FV consumption compared to the recommended level. Of the total respondents, only 11.3% had higher FV consumption and also met the minimum requirement. Respondents who lived in rural areas, were farmers, and grew FV at home had the highest probability of increased FV consumption. Exposure to the MMC and CBC was also associated with an increase in FV consumption. Above all, those who had exposure to the MMSMC, who reported either having high level of perception (OR = 1.832, 95% CI 1.259–2.666) or ever heard or seen (OR = 1.770, 95% CI 1.264–2.479) or heard and seen (OR = 1.698, 95% CI 1.131–2.550) campaign information were more likely to have a substantial increase in FV consumption and meeting/exceeding the recommended level than those who were not exposed to these campaigns. Other associated factors include education, occupation and physical activity. Implementation of MMSMC can help the population meet the global recommended level of FV consumption. This study presents its novelty since it was the first to highlight influence of mass media campaigns on increased FV consumption in Asian population. This was also the first study that used data from a population-based longitudinal study. The study suggested that MMC and CBC should also be promoted together with support systems to increase their intensity to a level that can increase population FV consumption to meet the recommended level. Sociodemographic characteristics should be taken into account, and targeted media is needed to effectively reach specific sub-groups of the population.

## 1. Introduction

Low fruit and vegetable (FV) consumption is among the leading dietary risk factors for death from noncommunicable diseases globally, and is estimated to cause 1.7 million (2.8%) deaths worldwide [1]. Policy options to tackle low FV consumption is therefore an important component of a shift towards healthier and more sustainable diets, and thus reduce deaths from noncommunicable diseases, and promote country sustainable development.

Implementing mass media campaigns (MMC) about healthy dietary practices, including social marketing initiatives is one of recommended population-level interventions that can improve diets [2]. It is well evident that implementation of MMC can produce positive changes or prevent negative changes in health-related behaviors across large populations through routine use of existing media, such as television, radio, and newspapers [3].

For increasing FV consumption in particular, MMC was deemed to be cost-effective, even more so when people were provided with access to healthy foods or had health disorders for which changes in diet would be beneficial [3]. An Australian study estimated that national campaigns to increase FV intake prevent 3,626 disability adjusted life years (DALY) each year with corresponding cost savings of ∼AUS$125 million each year over the implementation costs (estimated at ∼AUS$2.5 million a year) [4]. FV campaigns, e.g., ‘*the 2&5’* and ‘*Go for 2&5®*’ ads (promoting two servings of fruit intake and five servings of vegetable intake) launched by the Department of Health in Western Australia were found to be effective. The campaigns generated an excellent response from people in terms of familiarity with the Go for 2&5® message [5]. The campaigns reportedly increased awareness of the recommended servings of FV, and showed a population net increase of 0.8 in the mean number of servings of FV consumed per day over three years [6].

Other examples include implementation of the Fruits & Veggies Campaign to promote FV sales and intake to mothers and teens in California and Virginia in the US [7]. The Campaign used integrated marketing communications to engage target audiences with creative and humorous content. The result showed about 20% of the participants with increased awareness of the FV consumption after the 2-year implementation. Another study evaluated the state-wide health communication campaign (namely “Celebrate Your Plate Campaign”) to promote increased FV consumption or readiness to increase FV consumption among Ohio adults eligible for the Supplemental Nutrition Assistance Program (SNAP) [8]. The Campaign used several channels (online and offline) and included supportive individual- and community-level programming to reach target audiences. The evaluation reportedly significant association between the campaign exposure and increased FV consumption and readiness of increased FV consumption among the audience.

Inadequate FV consumption is among the top five leading causes of deaths in the Thai population, with 21,650 attributable mortality in 2014 [9]. Only one in three Thais met global recommended level (at least 400 gram per day [10]) of FV consumption in 2018 [11]. In recent years, the Thai Government has implemented various MMC to inform people about food and nutrition and raising public awareness about healthy diets, including reducing consumption of foods high in fat/sugar/salt and increasing FV consumption [12]. One agency in particular, the Thai Health Promotion Foundation (ThaiHealth), created a number of mass media social marketing campaigns (MMSMC) on healthy diet. A recent campaign used the slogan: *‘2*:*1*:*1’* (2 servings of vegetables, one serving each of rice and meat) [13]. Other campaigns in Thailand include community-based campaigns (CBC) to promote FV consumption among people at district and subdistrict levels. The CBC are typically implemented by civil society organizations (CSO), community residents, and village health volunteers.

Given the evidence of the influence of MMC, it is imperative to understand the factors and facilitators for improving healthy diets and, in particular, adequate FV consumption. While the influence of MMC on diets has been demonstrated in some settings, there is scant evidence of this influence in the Thai context. Thus, this study aimed to investigate the association between implementation of MMC, MMSMC, and CBC with FV consumption and other factors, and whether the level of FV consumption meets the global recommended level in the Thai population. The specific objectives of the study were 1) to analyse changes in FV consumption of Thais, 2) to analyse association between level of exposure to FV campaigns and other factors (such as socio-demographic characteristics, health status, access to FV, and FV knowledge) and chance of increasing FV consumption (either reaching or not reaching the recommended level), and 3) to analyse association between of level of exposure to FV campaigns and other factors with chance of reaching the recommended level of FV consumption.

## 2. Materials and methods

### 2.1. Study design and population

This study used data from a nationally-representative household survey of the Thai population, namely ‘A Longitudinal Study on Fruit and Vegetable Eating Behaviors’ [11]. The data for this study drew from Round1 (baseline) (in 2018) and Round2 (in 2019). The survey was designed to provide representation of three age groups (6–14 years, 15–59 years, 60 years or older), gender (male, female), and place of residence (rural, urban). In this study a target age group for participants was 15 years or older who are set to answer questions about exposure to implementation of MMC, MMSMC, and CBC. The participants were divided into four age groups: 15–29 years, 30–44 years, 45–59 years and 60 years or older.

The study applied a multi-stage sampling design to obtain a nationally representative sample of persons age six years or older in the four geographic regions of Thailand (North, Central, Northeast and South) and Bangkok. The sampling process was conducted by the National Statistical Office—an agency of the Ministry of Digital Economy and Society, which is responsible for the national census.

The sampling frame was a hierarchical sampling structure in which households are nested within sampled province, district, and enumeration areas, respectively. Within each sampled districts from the nine selected provinces, EA were randomly chosen from a nationally representative sampling frame used in the National Statistical Office’s census. Twenty households were selected from each enumeration area. At the household level, all the household members who were present during field survey team visits were recruited. A replacement was not taken if the researcher failed to reach a household member. The sampling was conducted by the National Statistical Office, and the data collection was carried out by research team (led by SP and ST).

All procedures involving research study participants were approved by the Institutional Review Board of the Institute for Population and Social Research of Mahidol University (COA. No. 2018/02-070 and COA. No. 2019/06-200). Written informed consent was obtained from all subjects. For the subjects under 18 years the study obtained written consent from their parents or guardians before data collection.

### 2.2. Data collection

This study used the data collected in Rounds1 and 2 surveys, from the same households. The data from the household members age 15 years or older who participated in both Rounds1 and 2 were included in analysis.

A total of 3,720 households were sampled for the survey. In the first round, 3,670 households successfully participated in the survey, with a total of 6,713 respondents who completed the survey questionnaire (Table 1). In the second round, a total of 3,333 households successfully participated in the survey, with a total of 6,530 respondents who completed the survey questionnaire. In total, 4,649 respondents successfully completed both Round1 and 2 survey questionnaires. Face-to-face interviews were conducted with respondents by trained field data collectors.

**Table 1:** Number of sampled households and respondents (age 15 years or older) of the survey in Rounds1 and 2.

Region	Sampled Households	Number of households and respondents who completed the survey questionnaire				
		Round1 (2018)		Round2 (2019)		
		Households	Respondents	Households	Respondents	Respondents (in Rounds 1 and 2)
**Bangkok**	620	619	1,150	513	1,017	761
Central	660	637	1,178	581	1,197	783
North	720	707	1,235	707	1,218	844
Northeast	920	908	1,610	853	1,639	1,140
South	800	799	1,540	734	1,459	1,121
**Total**	**3,720**	**3,670**	**6,713**	**3,333**	**6,530**	**4,649**

### 2.3. Study variables

#### 2.3.1. Daily amount of FV consumption

The respondents were asked ‘*How often*, *in the past week*, *did you eat the following fruits/vegetables*?’ They were told to indicate ‘*Frequency of fruit/vegetable consumption (per week)*,’ ‘*Number of times a day consumed*’ and ‘*Average amount of fruit/vegetable consumption (each sitting)*’ for each fruit and vegetable group (S1 File).

Servings in this study were determined based on the standard serving adapted from the Thailand Nutrition Flag [14]. Detailed description of one standard serving of FV are presented in an earlier study [15]. Responses to the frequency and quantity were multiplied to calculate the average amount consumed in the past week. Adequate level of FV intake in this study was defined as consumption of at least 400 grams of FV per day (according to WHO’s global recommendation) [10].

Data from 3,010 respondents who reported eating below the recommended level in Round1 (baseline) were used to analyze changes in their consumption at the time of Round2, one year later. The respondents who already met the recommended level of FV consumption in Round1 were excluded from this study.

#### 2.3.2. Implementation of MMC, MMSMC, and CBC

This study examined FV consumption of Thais exposed to MMC conducted by the Thai Government, MMSMC by ThaiHealth, and CBC conducted by CSO.

The government concentrated on broadcasting MMC nationally to improve healthy diets by reducing consumption of foods high in fat/sugar/salt and increasing FV consumption. From time to time, specific MMC on FV were launched, such as ‘*eat five colors a day’* (to promote eating a variety of FV), ‘*five servings per day*’ (to promote adequate FV consumption), and ‘*eat safe fresh FV’* (to promote how to properly wash FV) [12]. The government’s campaign was mainly conducted with one-way communication to give factual information about benefits of a healthy diet, and guidelines for proper eating behavior. The messages were delivered via billboards, flyers/pamphlets, TV, and radio.

ThaiHealth focused on modifying the social structure and environment to make healthier choices easier choices, and used MMSMC as the major approach to influence health behavior of the population. These campaigns included different types and varying intensities of communication strategies through mass media, such as TV/radio, websites, social media, public relations events, and print material. The most recent ThaiHealth campaign used the slogan ‘*2*:*1*:*1’* that promotes single-meal consumption which includes two servings of vegetables and one serving each of rice and meat. This campaign was managed in conjunction with other activities such as Valentine’s Day public events on the theme of ‘*For the Love of Vegetables*,’ and on ‘*Promote Vegetables as a Priority’* to advocate for FV consumption of at least 400 grams a day [13].

CBC in Thailand typically aimed to advocate promotion of FV-related behaviors, such as adequate FV intake and healthy food environments such as home and community gardening and FV safety. Some campaigns delivered multiple messages to target communities, and FV consumption was integrated as part of broader healthy diet promotion. The target audience for the CBC include key influencers at the village, subdistrict, and district levels. The CBC campaigns were typically implemented by CSO, in collaboration with local residents and village health volunteers.

Each respondent in the longitudinal survey was asked the following questions: *“Have you ever heard mass media campaigns regarding adequate FV consumption*, *launched by the government*?*”; “Have you ever heard the ‘2*:*1*:*1 campaign’*?*”;* and lastly *“Have you ever heard campaigns regarding adequate FV consumption in your home community or an area where you were staying*?*”*

The respondent’s response from Rounds1 and 2 were used for analysis. In Round1, response categories were: ‘very low,’ ‘low,’ ‘high,’ and ‘very high’ levels of exposure to the campaigns. In order to simplify the analysis, these categories were recoded into two summary levels: ‘low (including ‘low’ and ‘very low’),’ and ‘high (including ‘high’ and ‘very high’). In Round2, response categories were slightly changed to: ‘never heard/seen,’ and ‘ever heard/seen.’

#### 2.3.3. Other variables

The study analyzed socio-demographic characteristics of the sample, as well as data on health status, physical activity, access to FV, and knowledge about recommended amount of FV consumption. Data from Round1 were used for these variables since the sample in both rounds comprise the same individuals.

*Socio-demographic characteristics*: Each respondent was asked to report gender, age, marital status, place of residence, education, occupation, and income.

*Health status*: Each respondent was asked if s/he currently has any chronic condition or disease. Response categories were ‘*yes*’ and ‘*no*.’

*Physical activity*: Each respondent was asked, “*Do you engage in physical activity for at least 30 minutes per day and at least 3 days per week (*or totaling 210 minutes a week of moderate intensity activity, e.g., brisk walking, running, aerobics, competitive games, and sports*)*?*”* Response categories were *‘yes’* and *‘no*.’

*Access to FV*: Each respondent was asked, “*In the past month*, *how did you obtain fresh FV most often*?*”* Response categories were ‘*someone else bought for me*,*’ ‘bought by myself*,*’ ‘home garden*,’ and ‘*picked from somewhere else/asked to borrow from neighbors*.’

#### 2.3.4. Data analysis

Multivariate statistics were used to test for significance of the association between increased FV consumption between the two rounds of the survey and each of the independent variables (i.e., exposure to MMC, MMSMC and CBC, sociodemographic characteristics, and health and FV-related variables). The tests include Chi-square (χ2).

Binary logistic regression analysis was used to examine the association between each independent variable with increased FV consumption (including both increasing and meeting the recommended amount, and increasing but failing to meet the recommended amount) with increased FV consumption and meeting the recommended amount. Odds ratios (ORs) were calculated, and estimates are presented with a 95% confidence interval (CI). A relationship with a *p* value of 0.05 or less was considered statistically significant.

## 3. Results

### 3.1. Changes in FV consumption

Of the 3,010 respondents, 53.0% had an increased level of FV consumption, but still below the minimum recommended amount (400 grams per day) in Round2 (Table 2). Only 11.3% had higher FV consumption and also met the minimum daily requirement. Nearly 36% consumed less than or the same amount as in Round1. More detailed descriptions of participant characteristics in Round2 are provided in S1 Table.

**Table 2:** Changes in frequency (N) and percentage (%) of FV consumption between Round1 (2018) and Round2 (2019).

Changes in FV consumption from Round1 to Round2	N	%
Eat less	1,065	35.4
Eat the same amount	9	0.3
Eat more but less than 400 g per day	1,596	53.0
Eat more and at least 400 g per day	340	11.3
**Total**	**3,010**	**100.0**

Table 3 shows the association between level of exposure to FV campaigns and other factors with increased FV consumption. Statistically significant differences were found in relation exposure to the MMC, MMSMC and CBC, place of residence, occupation, income, physical activity, and how FV were accessed.

**Table 3:** Chi-square test of association between exposure to FV campaigns and other factors, and changes in FV consumption.

Variables	People who ate less FV than 400 grams per day in Round1			
	(N = 3,010)			
	Respondents who ate more FV in Round2 (regardless of amount) (%)	χ^2^	Respondents who ate more FV and at least 400 g per day in Round2 (%)	χ^2^
**Gender**		**		
Male (N = 1,088)	61.0		10.1	
Female (N = 1,922)	66.2		12.0	
**Age (years)**		**		
15–29 (N = 402)	58.7		9.7	
30–44 (N = 522)	60.2		10.7	
45–59 (N = 1,044)	68.5		12.9	
60 years or older (N = 1,042)	64.4		10.6	
**Marital status**		**		
Single (N = 437)	57.4		8.9	
Married (N = 2,007)	65.7		12.3	
Widowed/divorced/separated (N = 566)	64.7		9.7	
**Place of residence**		***		*
Urban (N = 1,637)	59.9		10.2	
Rural (N = 1,373)	69.6		12.6	
**Education**		**		
Primary school or lower (N = 1,788)	66.5		12.1	
Secondary school (N = 977)	60.0		11.0	
Bachelor’s degree or higher (N = 245)	65.7		6.9	
**Occupation**		***		**
Unemployed (N = 1,153)	64.1		10.7	
Government job (N = 100)	69.0		15.0	
Company hire (N = 214)	50.5		3.3	
Own business (N = 587)	62.0		10.7	
Wage laborer (N = 392)	62.8		14.0	
Farmer (N = 564)	72.7		13.7	
**Income (baht per month)**		*		*
Less than 10,000 (N = 2,066)	65.5		12.1	
10,000–19,999 (N = 601)	59.1		8.5	
20,000 or above (N = 343)	66.2		11.4	
**Having chronic condition or disease**				
Yes (N = 1,265)	63.1		11.1	
No (N = 1,745)	66.0		11.4	
**Physical activity**		*		**
Yes (N = 1,202)	67.0		13.3	
No (N = 1,808)	62.6		10.0	
**How obtained FV**		***		*
Someone else bought them (N = 1,077)	59.9		9.4	
Bought by myself (N = 1,219)	63.1		11.2	
Home garden (N = 579)	74.1		14.2	
Picked from elsewhere or borrowed from neighbors (N = 135)	68.9		15.6	
**Exposure to MMC (Round1 / Round2)**		***		**
Low / Never heard (N = 1,095)	60.4		9.3	
Low / Ever heard (N = 541)	69.7		13.1	
High / Never heard (N = 763)	60.0		10.2	
High / Ever heard (N = 611)	72.0		14.6	
**Exposure to CBC (Round1 / Round2)**		***		**
Low / Never heard (N = 1,095)	60.5		9.9	
Low / Ever heard (N = 541)	75.4		15.1	
High / Never heard (N = 763)	66.6		12.0	
High / Ever heard (N = 611)	76.8		16.3	
**Exposure to MMSMC (Round1 / Round2)**				***
Low / Never heard (N = 1,095)	62.6		8.4	
Low / Ever heard (N = 541)	66.5		14.0	
High / Never heard (N = 763)	63.1		13.2	
High / Ever heard (N = 611)	67.9		13.3	

χ^2^ = Pearson Chi-Square.

*p≤0.05

**p≤0.01

***p≤0.001.

All variables (except having a chronic condition/illness and exposure to MMSMC) were associated with increased FV consumption. In particular, exposure to MMC (p≤0.01) and MMSMC (p≤0.001), and CBC (p≤0.01) was associated with achieving the recommended level of FV consumption. Other factors that have significant associations with consuming the recommended level of FV were place of residence (p≤0.05), occupation (p≤0.01), income (p≤0.05), physical activity (p≤0.01), and how FV were accessed (p≤0.05).

### 3.2. Regression of achieving the recommended level on exposure to implementations of FV campaigns and other associated factors

Tables 4 and 5 shows results from the binary logistic regression analysis. Model 1 presents an analysis of association between the studied factors and chance of increasing FV consumption (either reaching or not reaching the recommended level), compared to lower FV consumption. Model 2 presents an analysis of the association between the studied factors and chance of reaching the recommended level of FV consumption, compared to consuming less, the same, or higher amount of FV consumption (but still below the recommendation).

**Table 4:** Binary logistic regression of implementation of FV campaigns and other factors affecting increases in FV consumption (Model 1).

Variables	Respondents who ate more FV in Round2 (either meeting at least 400 g per day, or not) OR (95% CI)
**Gender (Reference = Male)**	
Female	1.189 (0.995–1.422)
**Age (Reference = 15–29)**	
30–44	0.830 (0.601–1.147)
45–59	1.044 (0.751–1.451)
60 years or over	0.852 (0.603–1.204)
**Marital status (Reference = Single)**	
Married	1.193 (0.911–1.561)
Widowed/divorced/separated	1.171 (0.849–1.617)
**Place of residence (Reference = Urban)**	
Rural	1.305 (1.106–1.540)**
**Education (Reference = Primary school or lower)**	
Secondary school	0.858 (0.699–1.053)
Bachelor’s degree or higher	1.154 (0.808–1.649)
**Occupation (Reference = Company hire)**	
Unemployed	1.621 (1.134–2.318)**
Government job	1.538 (0.907–2.607)
Own business	1.413 (1.018–2.162)*
Wage laborer	1.483 (1.018–2.162)*
Farmer	1.860 (1.267–2.731)**
**Income (baht per month) (Reference = Less than 10,000)**	
10,000–19,999	0.951 (0.754–1.201)
20,000 or above	1.279 (0.944–1.734)
**Having chronic condition or disease (Reference = Yes)**	
No	1.052 (0.883–1.252)
**Physical activity (Reference = No)**	
Yes	1.137 (0.969–1.334)
**How obtained FV (Reference = Someone else bought them)**	
Bought by myself	1.030 (0.850–1.248)
Home garden	1.374 (1.071–1.762)*
Picked from elsewhere or borrowed from neighbors	1.141 (0.761–1.709)
**Exposure to MMC (Round1 / Round2) (Reference = Low / Never heard)**	
Low / Ever heard	1.380 (1.084–1.758)**
High / Never heard	0.878 (0.691–1.115)
High / Ever heard	1.408 (1.076–1.844)*
**Exposure to CBC (Round1 / Round2) (Reference = Low / Never heard)**	
Low / Ever heard	1.430 (1.002–2.041)*
High / Never heard	1.249 (1.019–1.530)*
High / Ever heard	1.491 (1.053–2.111)*
**Exposure to MMSMC (Round1 / Round2) (Reference = Low / Never heard)**	
Low / Ever heard	1.124 (0.894–1.414)
High / Never heard	0.969 (0.755–1.244)
High / Ever heard	1.021 (0.774–1.346)
**Cox & Snell R** ^**2**^	**0.047**

*p≤0.05

**p≤0.01

***p≤0.001.

**Table 5:** Binary logistic regression of implementation of FV campaigns and other factors affecting increases in FV consumption and achieving the recommended level of consumption (Model 2).

Variables	Respondents who ate more FV and at least 400 g a day in Round2 OR (95% CI)
**Gender (Reference = Male)**	
Female	1.211 (0.920–1.595)
**Age (Reference = 15–29)**	
30–44	0.838 (0.493–1.425)
45–59	0.897 (0.532–1.514)
60 years or over	0.833 (0.481–1.443)
**Marital status (Reference = Single)**	
Married	1.254 (0.799–1.967)
Widowed/divorced/separated	0.968 (0.569–1.645)
**Place of residence (Reference = Urban)**	
Rural	1.098 (0.860–1.400)
**Education (Reference = Primary school or lower)**	
Secondary school	0.862 (0.633–1.173)
Bachelor’s degree or higher	0.491 (0.261–0.923)*
**Occupation (Reference = Company hire)**	
Unemployed	2.785 (1.215–6.384)*
Government job	4.421 (1.682–11.621)**
Own business	2.839 (1.251–6.441)*
Wage laborer	3.812 (1.643–8.845)**
Farmer	3.078 (1.323–7.157)**
**Income (baht per month) (Reference = Less than 10,000)**	
10,000–19,999	0.777 (0.540–1.119)
20,000 or above	1.221 (0.782–1.905)
**Having chronic condition or disease (Reference = Yes)**	
No	0.939 (0.725–1.217)
**Physical activity (Reference = No)**	
Yes	1.296 (1.026–1.638)*
**How obtained FV (Reference = Someone else bought them)**	
Bought by myself	1.083 (0.796–1.472)
Home garden	1.231 (0.865–1.753)
Picked from elsewhere or borrowed from neighbors	1.450 (0.846–2.485)
**Exposure to MMC (Round1 / Round2) (Reference = Low / Never heard)**	
Low / Ever heard	1.226 (0.858–1.751)
High / Never heard	0.765 (0.522–1.122)
High / Ever heard	1.094 (0.742–1.612)
**Exposure to CBC (Round1 / Round2) (Reference = Low / Never heard)**	
Low / Ever heard	1.125 (0.718–1.762)
High / Never heard	1.032 (0.760–1.400)
High / Ever heard	1.095 (0.713–1.683)
**Exposure to MMSMC (Round1 / Round2) (Reference = Low / Never heard)**	
Low / Ever heard	1.770 (1.264–2.479)**
High / Never heard	1.832 (1.259–2.666)**
High / Ever heard	1.698 (1.131–2.550)*
**Cox & Snell R** ^**2**^	**0.027**

*p≤0.05

**p≤0.01

***p≤0.001.

Model 1 (Table 4) shows that respondents who lived in rural areas, were farmers, and grew FV at home had the highest possibility of increasing FV consumption. Exposure to the MMC and CBC was found to be associated with an increase in FV consumption.

For Model 2 (Table 5), respondents who reported either having high level of perception (OR = 1.832, 95% CI 1.259–2.666) or ever heard or seen (OR = 1.770, 95% CI 1.264–2.479) or seen and heard about (OR = 1.698, 95% CI 1.131–2.550) the MMSMC were significantly more likely to achieving the recommended level of FV consumption. However, implementations of the MMC and CBC did not significantly affect respondents’ consumption to reach the recommended level.

The results of Model 2 analysis also point to other associated factors. Those factors include education, occupation, and physical activity. The respondents with a bachelor’s degree or higher education had the lowest probability of reaching the recommended amount of FV consumption (compared to primary or lower educational attainment groups) (OR = 0.491, 95% CI 0.261–0.923). The respondents who worked for the government had the highest probability of meeting the recommended level (OR = 4.421, 95% CI 1.682–11.621). Participants who had regular physical activity were 1.3 times more likely to meet the recommended amount of FV consumption (p≤0.05).

## 4. Discussion

The results of the present study show that implementation of the population-wide media campaigns influenced improvements in FV consumption in the population exposed to the campaigns. Furthermore, the results demonstrated that a substantial increase in FV consumption to meet the global recommended level was significantly associated with exposure to the MMC including social marketing.

Respondents exposed to the MMSMC by ThaiHealth were more likely to have a substantial increase in FV consumption and reach the recommended level than those who were not exposed to these campaigns. There is a significant body of evidence on the effectiveness of this type of campaign on large populations. MMC, including social marketing, which focused on increasing FV consumption were found to be successful and even more so when they were implemented in conjunction with other food policies such as access to healthy foods [5, 6, 16–19]. Assessment of the “*Go for 2&5*®” FV social marketing campaign in Australia, for example, showed positive impact in terms of high population coverage and increased FV servings per day [6]. Other food-related MMSMC such as using signs and labels providing nutritional information at the point of purchase also increased the likelihood of people’s healthier food choice [18]. In addition, as part of ThaiHealth’s mandate which places high importance on promoting health in communities, local communities frequently get involved in the related activities and, as such, may increase intensity and range of exposure to the MMSMC to a wider population, especially locals. The MMSMC are also an effective way to change health behavior in other areas of health risk such as tobacco consumption, and that is well evident as a highly cost-effective intervention both in higher- and in lower- and middle-income countries [20, 21]. The MMSMC can reduce tobacco use prevalence and prevent its uptake [22]. Despite the good promise of MMSMC, a significant challenge for policy makers and implementers to overcome is how to enable more sustained implementation of the MMSMC. Support systems for sustained funding are needed so that people can be exposed repeatedly to maintain an adequate level of consumption.

Although implementation of the government’s MMC was found to be associated with increased FV consumption, it was not enough to influence people to consume at the recommended level. This may be due to the one-way communication strategy of the MMC. In comparison, the ThaiHealth’s approach included opportunities for more interaction and frequency of contact from the target audience to the campaign implementers. One-way communication generally aims to produce a predefined attitudinal change in the audience [23], through giving factual information or persuading/manipulating people to take a certain action. As such, government implementation could succeed in persuading or manipulating people to increase FV consumption in Round1, but fell short in reaching the recommended level in the following year. Therefore, creating more attractive factual messages specific to each target population is important. Support systems for funding, communication skills, knowledge, and progress tracking are strongly recommended so that people can be exposed repeatedly up to the intensity level that can change attitudes and behavior toward consuming adequate FV.

The CBC could increase FV consumption, but not yet at the recommended level. Despite a large and growing number of studies investigating the effectiveness of community-based interventions to promote healthy eating, evidence for beneficial effects of community awareness campaigns on healthy eating is still ambiguous. The review of the literature found that beneficial effects of community-based approaches, e.g., awareness campaigns on healthy diets, are only achieved when combined with other approaches such as changes of the school food environment [24–26]. In Thailand, CSO and community members play a key role in designing, implementing, and/or facilitating implementation of the community-based initiatives. Civil society, in particular, is usually driven by a self-interest that might be at odds with the nature of the issue, as well as the focus on a specific agenda. Accordingly, CSO priorities may limit the effectiveness of the CBC. Implementation challenges for community-based interventions are mainly resource constraints, intervention adaptation, and challenges and conflicts within the organizational culture [27, 28]. Validity of the interventions and the potential for long-term gains are also challenges that must be faced [29]. Despite these, developing and implementing sound interventions at a community level are still essential for policy change at the local level. This suggests that better planning and theorizing for future CBC for FV consumption might improve or help overcome those challenges. Appropriate methodological triage and development of potential interventions in a step-wise manner are important. The implementers and facilitators may also need to be more strategic and build strong partnerships to pool and maximize expertise, and make the best use of limited resources. Local government should be engaged in the implementation and contribution to support CBC, both at the organizational and community levels, including implementation, adaptation, and collaborative technical assistance.

The findings show that the substantial increase in FV consumption and meeting the recommended level differed by education. The lowest probability of meeting the recommended level was found among respondents with a bachelor’s degree or higher education. Although other studies found a positive effect of higher levels of schooling on improvements in health-related behaviors, including healthy diets [30–33], no studies found a significant influence of higher education on a substantial increase in FV consumption to meet the recommended level. The present findings might be explained by the fact that meeting the recommended level might need a higher level of effort and environmental support to help people substantially improved their diet. People with higher education typically work at an office and, as such, might face some obstacles to taking control of the available meal options at work and self-monitor FV consumption to meet the recommended level. The present study also found that company-hired persons were less likely to meet the recommended level of FV consumption. Therefore, creating an environment in which the target population group—especially in company-hired personnel—can have greater control over their own meals in order to meet the recommended level. Accordingly, targeted media is also needed to reach target populations because heterogeneous audiences might need heterogeneous messages to be persuasive.

Regular physical activity was positively associated with increased FV consumption to meet the recommended level of FV consumption. This may be because people with regular physical activity are more likely to have favorable attitudes and self-efficacy [34, 35] which are crucial elements to improve and sustain the desired behavior. As such, these elements may contribute to people’s awareness and confidence to improve other health behaviors such as FV intake. Therefore, promoting FV consumption in conjunction with physical activity could accelerate changes in people’s behavior toward consuming FV to meet the recommended level.

There are several advantages to this study. First, this study used data from a population-based longitudinal study, with high participation rates. That attribute increases the statistical power of the analysis and reduces risk for selection bias. This study went further than examining only the amount of FV intake. It also considered substantial increases in FV intake to meet the recommended level. The study also analyzed increased FV intake by perceived implementation of MMC, sociodemographic characteristics, and other FV-related factors.

This study is subject to limitations. The study relied on self-reported measures in which the accuracy of FV intake from a semi-food frequency questionnaire is variable and prone to participant recall bias. Moreover, inconsistency of respondents’ responses on exposure to the MMC and CBC in Rounds 1 and 2 may affect interpretation of the findings. The results on exposure to MMC were based on respondent’s perception, and that may be subject to bias. For example, there may be uneven reliability of a respondent’s view of false information due to fear of reporting actual perception or social desirability bias. In addition, some respondents may not be able to distinguish between the government, ThaiHealth, and community campaigns. Combining these data with data from in-depth analysis of MMC implementation would help monitor the actual effect of MMC over time. Future analysis of how people can sustain FV consumption at the recommended level is also needed to address factors which enable them to sustain heathy eating behaviors. That information would help inform effective population-based policy planning.

## 5. Conclusions

The results suggest that implementation of the MMSMC can help the population achieve the global recommended level of FV consumption. Implementation of MMC and CBC are also important to increase FV consumption. However, support systems are needed to help people to substantially increase their consumption to meet the WHO-recommended level. The findings also point out the importance of sociodemographic characteristics of audiences, in particular education and occupation. The findings from this study suggest that campaigns to increase in FV intake to meet the recommended level could be more effective if the campaigns are implemented in an environment which has support systems for sustainable implementation, with targeted media, and in conjunction with other food and nutrition policies. Future study is needed to analyze how people can sustain FV consumption at the recommended level in the long run.

## Supporting information

S1 File Questionnaire for ‘A Longitudinal Study on Fruit and Vegetable Eating Behaviors’. (DOCX)

S1 Table Summary of participants in Round2 by sociodemographic characteristics, health related behaviours, exposure to implementation of mass media campaign, and fruit and vegetable intake. (DOCX)
