# Supplementary material for: Implementing population-wide mass media campaigns: Key drivers to meet global recommendations on fruit and vegetable consumption
Source: PLoS One. 2022 Aug 17;17(8):e0273232. doi: 10.1371/journal.pone.0273232 (PMC9385052; doi:10.1371/journal.pone.0273232)
Supplement: S1 File — (DOCX) [file pone.0273232.s001.docx]

**Questionnaire for ‘A Longitudinal Study on Fruit and Vegetable Eating Behaviors’**

1. Gender

🞏 1. Male

🞏 2. Female

2. Age

🞏 1. 15 – 29 years

🞏 2. 30 – 44 years

🞏 3. 45 – 59 years

🞏 4. 60 years and above

3. Marital status

🞏 1. Single

🞏 2. Married

🞏 3. Widowed / Divorced / Separated

4. Place of residence

🞏 1. Urban

🞏 2. Rural

5. Education

🞏 1. No study

🞏 2. Primary school

🞏 3. Secondary school

🞏 4. Bachelor’s degree

🞏 5. Higher bachelor’s degree

6. Occupation

🞏 1. Unemployed

🞏 2. Government job

🞏 3. Company hire

🞏 4. Own business

🞏 5. Wage laborer

🞏 6. Farmer

7. Income (Thai baht per month)

_________________

8. Do you have any chronic condition or disease?

🞏 0. No

🞏 1. Yes

9. Do you engage in either of these physical activities? (Walk-running/cycling/aerobics/sports for at least 30 minutes a day and at least 3 times a week.)

🞏 0. No

🞏 1. Yes

11. In the past month, what is your regular access method to fruits and vegetables for home cooking?

🞏 1. Someone else buy them for me

🞏 2. Buy them myself

🞏 3. Home gardening

🞏 4. Pick them from somewhere else or ask neighbours

12A. Have you ever heard about implementation of mass media campaigns regarding adequate consumption of fruits and vegetables, launched by Thai Government? (Round 1)

🞏 1. Very low

🞏 2. Low

🞏 3. High

🞏 4. Very high

12B. Have you ever heard about implementation of mass media campaigns regarding adequate consumption of fruits and vegetables, launched by Thai Government? (Round 2)

🞏 1. Never heard/seen

🞏 2. Ever heard/seen

13A. Have you ever heard about implementation of campaigns regarding adequate consumption of fruits and vegetables in your residential community or an area where you live in? (Round 1)

🞏 1. Very low

🞏 2. Low

🞏 3. High

🞏 4. Very high

13B. Have you ever heard about implementation of campaigns regarding adequate consumption of fruits and vegetables in your residential community or an area where you live in? (Round 2)

🞏 1. Never heard/seen

🞏 2. Ever heard/seen

14A. Have you ever heard about implementation of 2:1:1 campaigns, launched by ThaiHealth? (Round 1)

🞏 1. Very low

🞏 2. Low

🞏 3. High

🞏 4. Very high

14B. Have you ever heard about implementation of 2:1:1 campaigns, launched by ThaiHealth? (Round 2)

🞏 1. Never heard/seen

🞏 2. Ever heard/seen

15. How often, in the past week, did you eat the following vegetables?

| **Group** | **Frequency of vegetable consumption**  **(per week)** | | | | | | | | **Number of times a day consumed** | **Average amount of vegetable consumption (each sitting)** | |
| --- | --- | --- | --- | --- | --- | --- | --- | --- | --- | --- | --- |
|  | None | 1  day | 2  days | 3  days | 4  days | 5  days | 6  days | 7  days |  | Sample of  one fruit  serving* | Number of  serving(s)  (rice-serving  spoon/ladle) |
| 1. Roots (such as carrot, radish, fingerroot, onion, red onion, ginger, galangal)  *not including sweet potatoes |  |  |  |  |  |  |  |  |  |  |  |
| 2. Stems and stalks (such as celery, lentils, green/spring onion, garlic chives, morning glory, water mimosa) |  |  |  |  |  |  |  |  |  | 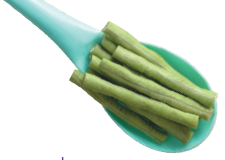 |  |
| 3. Leaves (such as cabbage, kale, spinach, levy gourd, Chinese cabbage (pak-choi, pe-tsai), lettuce, Baegu, Senna siamea, white popinac) |  |  |  |  |  |  |  |  |  |  |  |
| 4. Flowers/flower buds with stems/stalks (such as asparagus; broccoli, broccoli raab; Chinese broccoli, cauliflower, green cauliflower, Sesbania grandiflora, Sesbania flower, pumpkin flower, banana blossom, Siamese neem) |  |  |  |  |  |  |  |  |  |  |  |
| 5. Fruits (such as cucumber, zucchini, winter melon, green  eggplant, eggplant, bitter melon, pumpkin, tomato, twisted cluster bean, sweet/bell - peppers,  green/red/yellow) |  |  |  |  |  |  |  |  |  |    |  |

* Image from Healthy Eating Guidelines (2007) from the Bureau of Nutrition, Department of Health, Ministry of Public Health, Thailand

16. How often, in the past week, did you eat the following fruit?

| **Group** | **Frequency of vegetable consumption**  **(per week)** | | | | | | | | **Number of times a day consumed** | **Average amount of vegetable consumption (each sitting)** | |
| --- | --- | --- | --- | --- | --- | --- | --- | --- | --- | --- | --- |
|  | None | 1  day | 2  days | 3  days | 4  days | 5  days | 6  days | 7  days |  | Sample of  one fruit  serving* | Number of  serving(s)  (rice-serving  spoon/ladle) |
| 1. Very small-sized fruits (such as grapes, longan, lychee, Jamaican cherry, star gooseberry, Burmese grapes, strawberry, tamarind). |  |  |  |  |  |  |  |  |  |    |  |
| 2. Small-sized fruits (such as rambutan, mangosteen, Marian plum, Sapodilla) |  |  |  |  |  |  |  |  |  |    |  |
| 3. Small-medium-sized fruits (such as cultivated banana, tangerine, apple, pear, star apple) |  |  |  |  |  |  |  |  |  |      |  |
| 4. Large-medium-sized fruits (such as Cavendish banana, guava, mango, custard apple) |  |  |  |  |  |  |  |  |  | 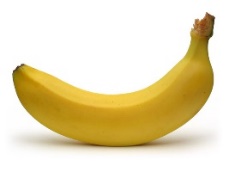  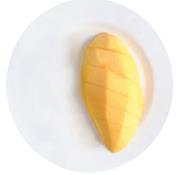 |  |
| 5. Large-sized fruits (such as watermelon, papaya, pineapple, cantaloupe, coconut) |  |  |  |  |  |  |  |  |  |    |  |

* Image from Healthy Eating Guidelines (2007) from the Bureau of Nutrition, Department of Health, Ministry of Public Health, Thailand
