# Supplementary material for: Implementing population-wide mass media campaigns: Key drivers to meet global recommendations on fruit and vegetable consumption
Source: PLoS One. 2022 Aug 17;17(8):e0273232. doi: 10.1371/journal.pone.0273232 (PMC9385052; doi:10.1371/journal.pone.0273232)
Supplement: S1 Table — (DOCX) [file pone.0273232.s002.docx]

**S1 Table** Summary of participants in Round2 by sociodemographic characteristics, health related behaviours, exposure to implementation of mass media campaign, and fruit and vegetable intake

| **Variables** | **N** | **%** |
| --- | --- | --- |
| Gender  Male  Female | 1,088  1,922 | 36.1  63.9 |
| Age (years)  15-29  30-44  45-59  60 years or older | 402  522  1,044  1,042 | 13.4  17.3  34.7  34.6 |
| Marital status  Single  Married  Widowed/divorced/separated | 437  2,007  566 | 14.5  66.7  18.8 |
| Place of residence  Urban  Rural | 1,637  1,373 | 54.4  45.6 |
| Education  Primary school or lower  Secondary school  Bachelor’s degree or higher | 1,788  977  245 | 59.4  32.5  8.1 |
| Occupation  Unemployed  Government  Company hire  Own business  Wage laborer  Farmer | 1,153  100  214  587  392  564 | 38.3  3.3  7.1  19.5  13.0  18.7 |
| Income (baht per month)  Less than 10,000  10,000 – 19,999  20,000 or above | 2,066  601  343 | 68.6  20.0  11.4 |
| Having chronic condition or disease  Yes  No | 1,265  1,745 | 42.0  58.0 |
| Physical activity  Yes  No | 1,202  1,808 | 39.9  60.1 |
| How obtained FV  Someone else bought them  Bought by myself  Home garden  Picked from elsewhere or borrowed from neighbors | 1,077  1,219  579  135 | 35.8  40.5  19.2  4.5 |
| Exposure to MMC  Never heard  Ever heard | 1,858  1,152 | 61.7  38.3 |
| Exposure to CBC  Never heard  Ever heard | 2,578  432 | 85.6  14.4 |
| Exposure to MMSMC  Never heard  Ever heard | 2,138  872 | 71.0  29.0 |
| FV intake per day  <400 grams per day  ≥400 grams per day | 2,670  340 | 88.7  11.3 |
| Total | 3,010 | 100.0 |
